# Anterior prostate biopsy at initial and repeat evaluation: is it useful to detect significant prostate cancer?

**DOI:** 10.1590/S1677-5538.IBJU.2014.0234

**Published:** 2015

**Authors:** Pietro Pepe, Michele Pennisi, Filippo Fraggetta

**Affiliations:** 1 Unità Urologia, Ospedale Cannizzaro, Catania, Italy; 2 Unità Patologia, Ospedale Cannizzaro, Catania, Italy

**Keywords:** Prostatic Neoplasms, Prostate, Biopsy

## Abstract

**Purpose:**

Detection rate for anterior prostate cancer (PCa) in men who underwent initial and repeat biopsy has been prospectively evaluated.

**Materials and Methods:**

From January 2013 to March 2014, 400 patients all of Caucasian origin (median age 63.5 years) underwent initial (285 cases) and repeat (115 cases) prostate biopsy; all the men had negative digital rectal examination and the indications to biopsy were: PSA values > 10 ng/mL, PSA between 4.1-10 or 2.6-4 ng/mL with free/total PSA≤25% and ≤20%, respectively. A median of 22 (initial biopsy) and 31 cores (repeat biopsy) were transperineally performed including 4 cores of the anterior zone (AZ) and 4 cores of the AZ plus 2 cores of the transition zone (TZ), respectively.

**Results:**

Median PSA was 7.9 ng/mL; overall, a PCa was found in 180 (45%) patients: in 135 (47.4%) and 45 (36%) of the men who underwent initial and repeat biopsy, respectively. An exclusive PCa of the anterior zone was found in the 8.9 (initial biopsy) vs 13.3% (repeat biopsy) of the men: a single microfocus of cancer was found in the 61.2% of the cases; moreover, in 7 out 18 AZ PCa the biopsy histology was predictive of significant cancer in 2 (28.5%) and 5 (71.5%) men who underwent initial and repeat biopsy, respectively.

**Conclusions:**

However AZ biopsies increased detection rate for PCa (10% of the cases), the majority of AZ PCa with histological findings predictive of clinically significant cancer were found at repeat biopsy (about 70% of the cases).

## INTRODUCTION

Prostate cancer (PCa) has become the most common tumor in men (1), but in about 50% of the cases diagnosed by a screening protocol an indolent PCa can be found increasing the risk of overtreatment (2); active surveillance program (AS) has been introduced in clinical practice (3) to reduce the number of unnecessary definitive treatment, but certain criteria to select aggressive PCa in early stages and in the presence of minimal PCa biopsy histological findings are still missing (4). Recently, multiparametric magnetic resonance imaging (mMRI) (5) and targeted MRI imaging/ultrasound fusion-guided biopsy (6, 7) have dramatically improved biopsy accuracy to detect significant PCa (8); however, the majority of urological Centers are still no Table to perform mMRI. In definitive, hoping that mMRI could be introduced routinely in clinical practice, the extended (EPBx) and saturation biopsy (SPBx) schemes performed at initial and repeat prostate biopsy should be improved; in this respect, repeat biopsy mMRI has found a significant percentage of cancers located in the anterior zone of the gland (about 20-30% of the cases) that would have been missed by standard SPBx (8, 9).

In our study, detection rate for PCa with a prostate biopsy scheme that included anterior zone cores in men who underwent initial and repeat procedure has been prospectively evaluated.

## MATERIALS AND METHODS

From January 2013 to March 2014, 400 patients all of Caucasian origin and between the ages of 47 and 75 years (median 63.5 years) underwent initial (285 cases) and repeat (115 cases) prostate biopsy; all men had negative digital rectal examination and the indications to biopsy were (10): PSA values>10ng/mL, PSA between 4.1-10 or 2.6-4ng/ mL with free/total PSA≤25% and ≤20%, respectively. In case of initial EPBx and repeat SPBx a median of 22 (range: 21-23) and 31 (range: 26-40) cores were performed: 9 (EPBx) vs 12 cores (SPBx) in the peripheral zone (PZ) of each lobe (apex, median zone and base of the gland) beginning parasagittally to reach the outer edges of the gland (lateral margins); in addition 4 cores of the anterior zone (AZ) and 4 cores of the AZ plus 2 cores of the transition zone (TZ) were sampled at initial and repeat biopsy, respectively. The procedure was done transperineally (11) using a tru-cut 18 gauge needle (Bard; Covington, GA), a GE Logiq 500 PRO ecograph (General Electric; Milwaukee, WI) supplied with a biplanar transrectal probe (5-6.5 MHz) under sedation and antibiotic prophylaxis (one tablet of levofloxacin for 3 days beginning the day before biopsy). All patients signed an informed consent form which, in addition to the biopsy-related complications, explicitly reported the risk of diagnosing a clinically non significant PCa when AZ cores are taken especially in case of initial biopsy. Detection rate and histological findings of PCa located in the PZ, AZ, TZ and PZ plus AZ were prospectively evaluated; moreover, quantitative biopsy histology was recorded (12). For statistical analysis the t Student's-test was used; a p value<0.05 was considered statistically significant.

## RESULTS

Median PSA was 7.9ng/mL (range: 2.858ng/mL): 50 (12.5%) had PSA>10ng/mL, 330 (82.5%) between 4-10 and 20 (5%) between 2.6-4ng/mL, respectively. Overall, a cT1c stage PCa was found in 180/400 (45%) patients: in 135 (47.4%) and 45 (36%) of the men who underwent initial and repeat biopsy, respectively. In the remaining 220 patients an intraepithelial prostatic neoplasia (HGPIN), an atypical small acinar proliferation (ASAP), a chronic prostatitis and a normal parenchyma was found, in 18 (4.5%), 3 (0.7%), 29 (7.2%) 170 (42.5%) cases, respectively. Overall, clinical parameters and histological findings in the presence of PCa are listed in Table-1. In detail, in case of initial biopsy the highest percentage of PCa was found in the PZ (71.2% of the cases) of the gland; moreover, the percentage of cancer detected in the PZ plus AZ increased from 13.3% (18/135 cases) to 44.5% (20/45 cases; p=0.0001) in patients submitted to initial and repeat biopsy, respectively. An exclusive PCa of the anterior zone was found in 8.9 (12/135 cases) vs 13.3% (6/45 cases) (p=0.36) of the men who underwent initial vs repeat biopsy, respectively; the histological findings of AZ cancers in comparison with the PCa located in the PZ and/or PZ plus AZ demonstrated a significantly lower value (Table-1) of positive cores (1 cores vs 6.5 and 7.5 cores; p=0.0001), GPC (greatest percentage of cancer: 14% vs 55% and 65%; p=0.0001) and TPC (total percentage of cancer: 1% vs 8% and 12%; p=0.017). The highest percentage of microfocus of cancer (one positive core with Gleason score of 6 and GPC≤5%) (12) was found in the AZ (11 patients equal to 61.2% of the cases); on the other hand, in 7 of 18 AZ PCa the biopsy histology was predictive of significant cancer (GS≥6 and GPC>50%) in 2 (28.5%) and 5 (71.5%) men who underwent initial and repeat biopsy, respectively. Finally, AZ cores found a statistically significantly higher percentage of PCa in comparison with TZ cores (6 vs 1 equal to 13.4% vs 3% of the cases, respectively; p=0.003).

**Table 1:** clinical and histological findings in the 180 patients (pts) with prostate cancer (PCa).

	Overall PCa	PZ PCa	AZ PCa	PZ + AZ PCa	TZ PCa
Clinical and histological parameters	180 pts (100%)	126 pts (71.2%)	18 pts (10%)	35 pts (19.5%)	1 pts (0.5%)
**PSA ng/mL (range)**	8.9 (2.8-58)	7.4 (2.8-19)	11.5 (5.3-21)	12.4 (5.2-60)	12.5 (12.5)
**Initial biopsy**	135 (75%)	105 (77.8%)	12 (8.9%)	18 (13.3%)	-
**Repeat biopsy**	45 (25%)	19 (42.2%)	6 (13.4%)	20 (44.4%)	1 (3%)
**Median GS (range)**	6.4 (6-8)	6.4 (6-9)	6.1 (6-7)	6.5 (6-8)	6 (6)
**Median GPC (range)**	56% (5-100%)	65% (5-100%)	14% (5-90%)	55% (5-100%)	5% (5%)
**Median TPC (range)**	8% (1-72%)	8% (1-60%)	1% (1%)	12% (1-72%)	1% (1%)
**No of positive cores (range)**	6.5 (1-23)	7 (1-19)	1 (1-2)	7.5 (1-23)	1 (1)
**Microfocus of PCa** *	34 (18.9%)	22 (17.5%)	11 (61.2%)	-	1 (100%)

**PZ =** Peripheral Zone; **AZ =** Anterior Zone; **TZ =** Transition Zone; **GS =** Gleason score; **GPC =** Greatest Percentage of Cancer; **TPC =** Total Percentage of Cancer;

* Microfocus of PCa = 1 positive core with GS of 6 and GPC≤5%.

Overall, side effects following prostate biopsy occurred in 36.2% (140/400) of patients: 105 (26.2%) cases of hemospermia, 42 (10.5) of acute urine retention and 36 (9%) of hematuria; none needed hospital admission; moreover, all patients had a grade I of the Clavien-Dindo complications scale (13).

## DISCUSSION

Aggressive screening and prostate needle biopsy protocols have been successful in early detection of low-volume tumors increasing the incidence of anterior-predominant prostate cancers especially in patients submitted to repeat biopsy (14–21). Anterior tumors are less likely to be palpable, are not easily visualized by imaging and may require more biopsy sessions to establish a diagnosis (14); moreover, even when diagnosed, biopsies usually yield fewer involved cores and less total tumor length, making the assessment of cancer volume difficult (18–20). However, recently Sundi et al. (9, 22) reported a higher prevalence of AZ cancers in African American men with very low-risk PCa (59% of the cases), and still today few data are available regarding the incidence and clinical relevance of the AZ PCa. Bott et al. (14) have shown that about one fifth of all index cancers were located anterior to the prostatic urethra; Hikmat et al. (15) in radical prostatectomy specimen demonstrated that 15% of PCa were located in the AZ of the gland and characterized by a superimposable grading and staging in comparison with PZ cancers. In the last years, mMRI and MRI imaging/ultrasound fusion-guided biopsy have increased the detection of PCa (5–7, 21) located in about 20-30% of the cases in the anterior zone of the gland; in this respect, transperineal prostate biopsy demonstrated a better accuracy in comparison with transrectal approach in the diagnosis of AZ cancers (23).

In our series, overall detection rate for cancer located in the anterior zone was equal to 10% (18/180 cases) in initial (8.9% of the cases) vs repeat (13.4% of the cases) prostate biopsy (p=0.36). In detail, 11/18 (61.2%) AZ PCa were characterized by the presence of a single microfocus of PCa at risk for clinically indolent PCa; on the other hand, 7/18 clinically significant AZ PCa (GS≥6 and GPC>50%) would have be missed through a standard biopsy scheme (2 vs 5 cases at initial vs repeat biopsy, respectively). In definitive, the AZ biopsies increased detection rate for PCa allowing to reduce the prostate biopsy false negative rate and to characterize the histological findings of the cancer; in addition, in case of repeat SPBx biopsy AZ cores found a statistically significantly higher percentage of significant PCa in comparison with TZ cores (13.4 vs 2% of the cases) (p=0.003).

Regarding our results, some limitations should be reported. Firstly, the true incidence of AZ PCa should be evaluated in the entire specimen of the prostate; secondly, the clinical relevance of the AZ PCa remains unknown; finally, a greater number of cases should be evaluated.

In conclusion, although AZ biopsies increased overall detection rate for PCa (10% of the cases) the majority of AZ cancers with histological findings predictive of clinically significant PCa were found in case of repeat procedures (about 70% of the cases).
